# Amiodarone-Induced Hypothyroidism Presenting as Cardiorenal Syndrome

**DOI:** 10.1155/2012/161450

**Published:** 2012-05-19

**Authors:** Evan L. Hardegree, Robert C. Albright

**Affiliations:** ^1^Department of Medicine, Mayo Clinic, 200 First Street SW, Rochester, MN 55905, USA; ^2^Division of Nephrology and Hypertension, Mayo Clinic, 200 First Street SW, Rochester, MN 55905, USA

## Abstract

Here we present the case of a 90-year-old man with chronic heart and renal failure who was admitted with what appeared to be a simple heart failure exacerbation. However, further investigation led to the diagnosis of profound amiodarone-induced hypothyroidism as the cause of his acute decompensation, highlighting the importance of a broad differential diagnosis and thorough investigation.

## 1. Introduction

In patients with concurrent heart and renal failure (cardiorenal syndrome), common causes of decompensation may include myocardial ischemia, excessive salt ingestion, and medication noncompliance. However, there are many conditions that may tip this delicate balance. Hypothyroidism, which may result from infection, inflammation, or use of certain medications, is a condition that typically presents with symptoms such as fatigue, weight gain, cold intolerance, and constipation. While such symptoms may be starkly apparent in young, healthy patients, they may be attributed to age or comorbid conditions in the elderly and chronically ill. Hypothyroidism may also dramatically worsen underlying cardiac and renal dysfunction and manifest in ways virtually indistinguishable from typical causes of heart failure, making it essential to keep this condition in the differential diagnosis of acutely decompensated heart failure.

## 2. Case Report

**Figure 1:**
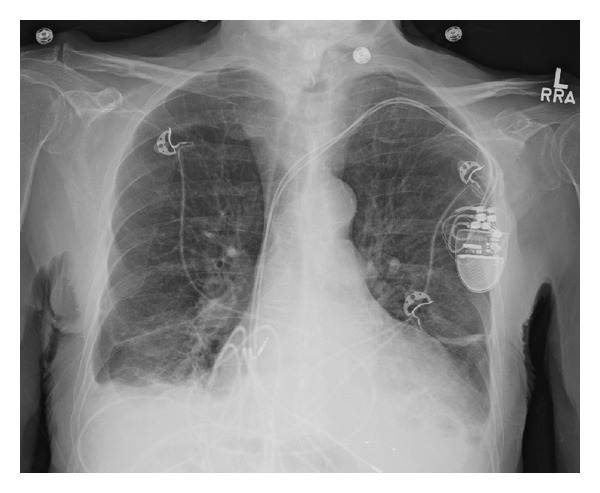
Chest X-ray demonstrating cardiomegaly, pleural effusions, and pulmonary vascular congestion.

**Figure 2:**
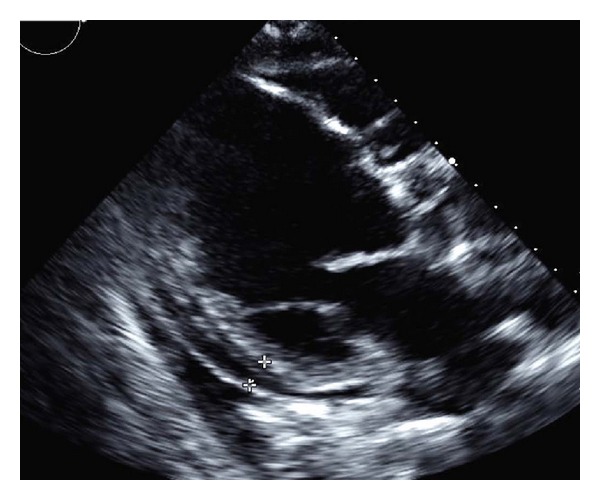
Transthoracic echocardiogram showing a moderate-sized pericardial effusion (marked).

A 90-year-old man presented for evaluation of a one-month history of fatigue and progressive abdominal and lower extremity edema. His history was notable for ischemic cardiomyopathy with a left ventricular ejection fraction (LVEF) of 15% with a dual-chamber pacemaker for cardiac resynchronization; paroxysmal atrial fibrillation and frequent ventricular ectopy requiring initiation of amiodarone 6 months prior (200 mcg/day); stage IV chronic kidney disease, not on dialysis. The patient reported full compliance with his medical heart failure regimen and denied any dietary indiscretion, alcohol intake, or anginal symptoms. His vital signs were unremarkable but his weight had increased by 4.6 kg. Examination revealed a regular heart rate of 60 beats/minute, jugular venous distension, a hepatojugular reflex, inspiratory crackles in the lung bases, and significant lower extremity pitting edema to mid-thigh level. The skin was notably dry. Thyroid exam was unremarkable. A chest X-ray revealed pulmonary edema and cardiomegaly (Figure 1). Electrocardiography revealed a paced rhythm at 62 beats/minute and cardiac biomarkers revealed no evidence of myocardial infarction. Laboratory studies included an N-terminal pro-B-type natriuretic peptide of 10,644 pg/mL (normal < 180) and serum creatinine of 3.7 mg/dL (GFR 16 mL/min), significantly worse than his baseline creatinine of 2.3 mg/dL. A transthoracic echocardiogram revealed an LVEF of 12% with global hypokinesis, a right ventricular (RV) systolic pressure of 44 mmHg, and a moderate-sized concentric pericardial effusion (Figure 2).

For treatment of decompensated heart failure, he was cautiously diuresed with furosemide. With this his pulmonary, abdominal, and peripheral edema improved, and his serum creatinine trended down to 3.2 mg/dL. However, he remained severely fatigued. Given this and the new pericardial effusion, serum thyroid studies were obtained, revealing a dramatically elevated thyroid-stimulating hormone of 187 mIU/L (normal 0.3 to 5.0), free thyroxine of 0.4 ng/dL (normal 0.8 to 1.8), and negative antithyroperoxidase antibodies. By contrast, his thyroid-stimulating hormone level was normal at 2.0 mIU/L immediately prior to initiation of amiodarone several months prior. He was diagnosed with amiodarone-induced hypothyroidism and started on low-dose oral levothyroxine (25 mcg/day), given his age, systolic heart failure, and history of ventricular dysrhythmias. His amiodarone was continued for the purpose of rhythm control. His fatigue and fluid status improved gradually over the next 4 days, and he was dismissed from the hospital.

## 3. Discussion

Amiodarone is a well-documented cause of thyroid dysfunction (hyper- or hypothyroidism) by virtue of the high iodine content as well as direct toxic effects on thyroid parenchyma. Hypothyroidism most commonly develops after 6–12 months of therapy, and while typical symptoms of hypothyroidism are seen (such as fatigue, weight gain, and cold intolerance), goiter is rare [1]. In elderly patients with chronic heart or renal failure, such symptoms may be overlooked or attributed to comorbid conditions. Furthermore, hypothyroidism causes a number of direct cardiovascular effects, including impaired contractility with reduced cardiac output, impaired diastolic relaxation, decreased heart rate, and increased peripheral vascular resistance (PVR). Additional findings may include pericardial effusion, nonpitting edema, QT prolongation, and ventricular ectopy [2]. Impaired ventricular function results from increases in PVR due to decreased release of endothelin-derived relaxation factor, reduced expression of beta adrenergic receptors resulting in blunted inotropic responses, and reduced expression of calcium-dependent enzymes involved in contraction and relaxation [2].

Cardiorenal syndrome is a term that applies to the pathophysiologic interactions in patients with both cardiac and renal dysfunction. This complex interplay was demonstrated in a 2006 systematic review of >80,000 patients with chronic heart failure, which demonstrated the concurrent existence of moderate-to-severe renal impairment in 29% of this population [3]. While it was once thought that renal impairment in heart failure was primarily due to reduced cardiac output, a number of additional factors have since been demonstrated. First, the reduced cardiac output causes neurohumoral adaptations, which activate the renin-angiotensin-aldosterone system. This results in salt and water retention and systemic vasoconstriction, causing increased afterload and reduced renal perfusion [4]. Additionally, RV dysfunction impairs LV filling via leftward shift of the interventricular septum (further reducing output) and also causes increased central venous pressure, which is transmitted to the renal veins, further worsening GFR [5]. Interestingly, reduction in RV filling pressures by diuresis has been shown to improve GFR, even without a concomitant improvement in cardiac output, likely by reduction in renal venous pressure as well as improvement of interventricular mechanics [6].

In summary, this case illustrates acutely decompensated heart and renal failure as an unusual manifestation of profound hypothyroidism, which was itself a complication of amiodarone therapy. This highlights the complex multisystem pathophysiology of cardiorenal syndrome, illustrates a potential side effect of amiodarone, and underscores the importance of a broad differential diagnosis and thorough workup.

## References

[1] Basaria S, Cooper DS (2005). Amiodarone and the thyroid. *American Journal of Medicine*.

[2] Klein I, Danzi S (2007). Thyroid disease and the heart. *Circulation*.

[3] Smith GL, Lichtman JH, Bracken MB (2006). Renal impairment and outcomes in heart failure. Systematic review and meta-analysis. *Journal of the American College of Cardiology*.

[4] Schrier RW, Abraham WT (1999). Hormones and hemodynamics in heart failure. *The New England Journal of Medicine*.

[5] Mullens W, Abrahams Z, Francis GS (2009). Importance of venous congestion for worsening of renal function in advanced decompensated heart failure. *Journal of the American College of Cardiology*.

[6] Testani JM, Khera AV, John SMGS (2010). Effect of right ventricular function and venous congestion on cardiorenal interactions during the treatment of decompensated heart failure. *American Journal of Cardiology*.

